# Endothelial Dysfunction in Kidney Transplantation

**DOI:** 10.3389/fimmu.2018.01130

**Published:** 2018-05-23

**Authors:** Héloïse Cardinal, Mélanie Dieudé, Marie-Josée Hébert

**Affiliations:** ^1^Research Centre, Centre hospitalier de l’Université de Montréal (CRCHUM), Montreal, QC, Canada; ^2^Canadian National Transplant Research Program, Montreal, QC, Canada; ^3^University of Montreal, Montreal, QC, Canada

**Keywords:** kidney transplantation, endothelial injury, apoptosis, necroptosis, alloantibodies, autoantibodies

## Abstract

Kidney transplantation entails a high likelihood of endothelial injury. The endothelium is a target of choice for injury by ischemia-reperfusion, alloantibodies, and autoantibodies. A certain degree of ischemia-reperfusion injury inevitably occurs in the immediate posttransplant setting and can manifest as delayed graft function. Acute rejection episodes, whether T-cell or antibody-mediated, can involve the graft micro- and macrovasculature, leading to endothelial injury and adverse long-term consequences on graft function and survival. In turn, caspase-3 activation in injured and dying endothelial cells favors the release of extracellular vesicles (apoptotic bodies and apoptotic exosome-like vesicles) that further enhance autoantibody production, complement deposition, and microvascular rarefaction. In this review, we present the evidence for endothelial injury, its causes and long-term consequences on graft outcomes in the field of kidney transplantation.

## Introduction

The endothelium plays an important role in vascular biology and regulation of renal function. Healthy endothelial cells are involved in vasodilation through nitric oxide (NO) release, which also inhibits platelet adhesion and aggregation, as well as leukocyte adhesion. Conversely, injured endothelial cells can develop a vasoconstrictive, pro-inflammatory, and procoagulant phenotype. Endothelial dysfunction is associated with traditional cardiovascular risk factors such as hypertension and diabetes, and it predicts atherosclerosis progression and cardiovascular events in the general population (1, 2). A large body of data shows that chronic kidney disease (CKD) is associated with endothelial dysfunction and/or apoptosis (3–7). Increased levels of circulating microparticles from apoptotic endothelial cells have been observed in patients with CKD (5, 6). Uremic solutes foster the production of these microparticles by endothelial cells (6), which in turn decrease NO release and impair endothelium-mediated dilation (5).

Kidney transplantation is the best mode of renal replacement therapy, improving both quality of life and life expectancy compared to dialysis (8, 9). Kidney transplantation restores renal function and improves endothelial function compared to dialysis (10, 11). Nevertheless, kidney transplantation entails a high likelihood of endothelial injury in the allograft. Given its intimate contact with the blood, the allograft endothelium is a target of choice for interactions with circulating inflammatory cells, cytokines, antibodies, and circulating pharmacological agents. First, a certain degree of ischemia-reperfusion injury (IRI) inevitably occurs in the immediate posttransplant setting and manifests as delayed graft function (DGF). IRI is associated with both tubular and endothelial damage, especially in the peritubular capillary network. Second, acute rejection episodes, whether T-cell or antibody-mediated, occur in 15–20% of kidney transplant recipients (2) and can involve the graft micro- and macrovasculature, leading to endothelial injury. This can alter renal blood flow and impair renal function, both acutely and on the long-term, favoring renal fibrosis and loss of renal function.

Last, the most commonly used immunosuppressive agents may have divergent impact on the graft endothelium after transplantation. Mycophenolic acid may protect the endothelium, but calcineurin inhibitors have an adverse impact on endothelial function and glucocorticoids can worsen endothelial function under physiological conditions and improve it in the presence of inflammation. While these topics are reviewed elsewhere (12–14), here we present the evidence for allograft endothelial injury that is associated with IRI, alloimmunity, and autoimmunity in kidney transplantation and describe its long-term consequences on graft outcomes.

## IRI Induces Endothelial Damage, Microvascular Rarefaction and Adverse Graft Outcomes

The kidney transplant procedure is inevitably associated with a certain degree of IRI. Donor type (deceased after cardiocirculatory arrest and neurologically deceased versus living) and length of cold and warm ischemic times are important risk factors for IRI (15). Clinically significant IRI manifests as DGF, or acute kidney injury (AKI) in the immediate posttransplant period. DGF is defined as the need for hemodialysis in the first week posttransplantation or failure of serum creatinine to decrease by more than 10% on the first three postoperative days, although other definitions have been used (16). Episodes of AKI are strong predictors of CKD in the general population (17–20). Similarly, DGF is associated with decreased long-term kidney graft survival (15, 21).

In the past decade, microvascular injury and endothelial dysfunction have emerged as pivotal elements in the pathogenesis of AKI (22, 23). In experimental models of IRI, renal perfusion in peritubular capillaries is compromised within minutes of unclamping (24). Endothelial dysfunction/injury and apoptosis compromise microcirculatory renal blood flow through decreased vasodilatory capacity, coagulation activation and the formation of microvascular thrombi, and increased rolling/adhesion of inflammatory cells (23, 25).

Because the regenerative capacity of endothelial cells in peritubular capillaries appears limited (26–28), microvascular damage occurring during an episode of AKI can lead to permanent peritubular capillary rarefaction (26–28). Loss of peritubular capillaries favors chronic hypoxia, leading to overexpression of hypoxia inducible factor 1 α (HIF-1α), favoring transcription of fibrogenic genes such as transforming growth factor β (TGF-β) and connective tissue growth factor (CTGF). It also favors accumulation of α-smooth muscle actin (α-SMA) positive myofibroblasts and production of fibrogenic mediators (22, 23, 28–31).

These phenomena eventually lead to progressive interstitial fibrosis/tubular atrophy and renal dysfunction in animal models and in human AKI (31, 32). In kidney transplant patients, peritubular capillary loss, assessed by comparing capillary density on 3-month posttransplant biopsies with capillary density on preimplantation biopsies, is strongly associated with interstitial fibrosis/tubular atrophy and graft dysfunction 1 year posttransplant (33). Recent animal studies using *in vivo* imaging and electron microscopy in murine models of AKI demonstrated a tight correlation between peritubular capillary injury, rarefaction, and renal fibrosis (34, 35). Ultrastructural changes to peritubular capillaries include focal widening of the subendothelial space, higher numbers of endothelial vacuoles, reduced numbers of fenestrations, and increased thickness of the basement membrane (35). Human kidney biopsy samples with progressive renal fibrosis showed strikingly similar ultrastructural findings. Taken together, these studies support the concept that IRI-associated AKI can lead to microvascular rarefaction which in turn plays a pivotal role in favoring interstitial fibrosis and long-term renal dysfunction in patients with native kidney disease and in kidney transplant recipients.

Kidneys from older donors are more susceptible to IRI and more likely to develop DGF (36–39). Increasing age and the presence of age-associated disorders, such as hypertension and type 2 diabetes, favor the accumulation of senescent cells within the vasculature and the kidney. Senescence is characterized by proliferative arrest, cell flattening and enlargement, and the production of an array of pro-inflammatory cytokines (IL-1α, IL-1β, IL-6, IL-8, matrix metalloproteiases, CTGF) known as senescence associated secretory phenotype (40). Senescent cells lack replicative potential and hence tissues with higher levels of senescent cells display lower repair capacity in the face of injury. Increased microvascular rarefaction and enhanced fibrosis have been observed following IRI in rodent models and in transplant patients (41, 42).

## Immune-Mediated Vascular and Endothelial Injury is Associated with Adverse Kidney Graft Outcomes

Acute rejection episodes occur in 15–20% of kidney transplant recipients (2). T-cell mediated rejections that involve the tubulointerstitial compartment are responsive to corticosteroid therapy and are reversible in a majority of cases. However, vascular involvement by the rejection process, also termed graft endarteritis, is an important risk factor for decreased long-term graft survival (43, 44). Endarteritis has classically been regarded as a T-cell-mediated phenomenon (45), with both alloreactive CD8+ and CD4+ T-cells infiltrating the allograft small-sized arteries (46). However, mounting evidence shows that endarteritis often clusters with microvascular inflammation (glomerulitis, peritubular capillaritis) and antibody-mediated damage (47). The deleterious impact of donor-specific alloantibodies (DSA) is illustrated by recent data showing that antibody-mediated rejection with endarteritis entails a worse prognosis than cell-mediated endarteritis alone (44). DSA can target class I human leukocyte antigen (HLA) molecules, which are constitutively expressed on all nucleated cells or class II HLA molecules, whose expression is restricted to B lymphocytes, antigen-presenting cells, and activated endothelial cells. Both class I and class II DSA can injure the endothelium though complement-dependent mechanisms and antibody-dependent cell-mediated cytotoxicity. DSA class I binding also affects the graft endothelium by inducing intracellular signaling which results in migration, proliferation, and resistance to apoptosis and complement-induced death that can have an impact on vascular remodeling and chronic allograft rejection (48). The effect of HLA class II DSA on cell signaling remains to be fully defined given constraints in experimental systems due to the restricted expression of their antigenic target. Although DSA IgG have long been recognized as deleterious to the allograft, the clinical relevance of DSA IgM remains unclear. Some studies have reported associations between IgM DSA, rejection, and decreased graft survival (49, 50).

Even when the allograft arteries are not involved, DSA can affect the graft microcirculation, which is associated with adverse outcomes. A threefold increase in the risk of graft loss was reported in DSA-positive cases of rejection affecting only the microcirculation compared to pure cell-mediated tubulointerstitial rejection (44). In another study, diffuse C4d staining in peritubular capillaries, which marks antibody-mediated complement activation through the classical pathway, was an independent adverse prognostic factor in patients with concurrent cell-mediated rejection, whether or not the graft arteries were involved (51). Hence, the presence of antibody-mediated damage to the microcirculation has prognostic implications in cases of acute rejection, whether or not graft arterial involvement is also present.

Donor-specific antibodies lead to adverse outcomes by injuring the graft endothelium. In patients with antibody-mediated rejection, elevated levels of endothelial transcripts including von Willebrand’s factor, caveolin 1, platelet/endothelial cell adhesion molecule, and E selectin have been found in the allograft tissue (52). The presence of circulating DSA and elevated endothelial transcripts in the allograft were associated with poorer long-term graft survival (52), even when evidence for complement activation was lacking (53). Taken together, these studies illustrate that endothelial injury in the allograft macro- or microvascular beds, especially when antibody-mediated, reduces graft survival. DSA-mediated endothelial damage can occur through both complement-dependent and independent pathways.

The persistence of cell- or antibody-mediated vascular and endothelial injury are closely linked with the development of allograft fibrosis and demise. In a swine kidney transplantation model, persistent inflammation in peritubular capillaries was strongly associated with the presence of proliferating α-actin positive myofibroblasts around peritubular capillaries and progression of interstitial fibrosis (54). Similar results were found in human kidney graft biopsies, where microvascular injury in peritubular capillaries (angioregression or capillary drop-out, apoptotic endothelial cells and lamination of the basement membrane) was strongly correlated with interstitial fibrosis, graft dysfunction, and proteinuria (55). Glomerular capillary loss was also associated with glomerular sclerosis and proteinuria.

Recent data suggest that, in addition to DSA, autoantibodies present at the time of transplantation or produced in the posttransplant period can accentuate and aggravate microvascular injury. This concept, coined “innate autoimmunity,” was put forward by Carroll and co-workers, as they identified the aggravating role of naturally occurring polyspecific IgM autoantibodies targeting non muscle myosin heavy chain and glycogen phosphorylase in models of intestinal and skeletal muscle IRI (56–58). They also showed that blockade of this autoantibody attenuated tissue damage in a model of cardiac IRI (59). Our group identified anti-perlecan/LG3 IgG autoantibodies of the IgG1 and IgG3 sub-types that target a cryptic C-terminal fragment of perlecan (LG3), as predictors of renal dysfunction in a murine model of renal IRI and in renal transplant patients (60). Elevated levels of anti-perlecan/LG3 at the time of transplantation are associated with an increased risk of vascular rejection and DGF (60, 61). In patients with DGF, anti-perlecan/LG3 autoantibodies predict reduced long-term renal function (60). Anti-perlecan/LG3 autoantibodies exhibit a specific tropism for the ischemic vasculature. In experimental models of vascular rejection and renal IRI, deposition of anti-perlecan/LG3 autoantibodies was significantly increased by ischemia (60, 61). This led to enhanced activation of the classical complement pathway, C4d deposition, peritubular capillary rarefaction, and renal fibrosis. Other autoantibodies, such as anti-angiotensin II type 1 receptors (AT1R) and anti-fibronectin antibodies, have been implicated in accentuation of renal acute vascular rejection and transplant glomerulopathy (38, 39). Anti-AT1R IgG autoantibodies also increase the risk of acute rejection and graft loss in renal transplant patients (62, 63). Ischemia was shown to increase the contractile activity of AT1R autoantibodies in isolated renal artery rings (64), suggesting the possibility of enhanced renal vasoconstriction and ischemia. Collectively, these reports add further support to the notion that renal microvascular injury, either induced by IRI, allo- or auto-antibodies or through synergistic interactions between these different factors, plays a major role in long-term renal allograft dysfunction.

## Endothelial Cell Death Contributes to Vascular Remodeling, Autoimmunity and Inflammation

The presence of dying renal cells in association with AKI or rejection episodes has been known for decades. However, the characterization of molecular pathways controlling regulated renal cell death responses is still an evolving field. Two major types of programmed cell death, apoptosis and necroptosis, have been characterized in association with AKI (23, 26, 65–73), although various death and inflammatory pathways such as ferroptosis and pyroptosis also likely contribute (74–76). Apoptosis can be initiated by two major initiating pathways: cell surface death receptors or mitochondrial outer membrane permeabilization. Both pathways converge on an effector phase triggered by caspases-3 activation and responsible for definitive degradation of key nuclear and cytoskeletal substrates leading to morphological changes such as membrane blebbing and nuclear condensation. However, ligation of death receptors, such as tumor necrosis factor or Fas, in conditions when caspases are inhibited can also activate a regulated form of necrosis referred to as “necroptosis” [reviewed in Ref. (77, 78)]. In this context, receptor-interacting protein 1 (RIPK1) phosphorylates RIPK3 and mixed lineage kinase domain-like protein (MLKL) leading to cell swelling and rupture (77, 78). Necroptosis is associated with an important inflammatory response secondary to the release of damage-associated molecular patterns and to the activation of the inflammasome leading to caspase-1 activation and release of IL-1β, IL-18, and IL-1α. Like necroptosis, pyroptosis is a type of regulated necrotic cell death. Pyroptosis is characterized by caspase-11/gasdermin D-dependent plasma membrane rupture, is highly pro-inflammatory, and has a unique feature: the caspase-1 dependent maturation of pro-inflammatory cytokines in a multiprotein complex called the inflammasome during the cell death process (71, 75).

Apoptosis has classically been considered as an inert or anti-inflammatory type of cell death, responsible for the physiological turnover of multiple cell types. During apoptosis, caspase activation inactivates mitochondrial DNA-induced type I interferon secretion and oxidizes danger signals. This inactivates danger associated molecular patterns (DAMP) molecules and prevents the development of an innate immune response to apoptotic cells (79). Effector caspase activation also leads to the release of chemotactic factors that recruit phagocytes and enhance the clearance of apoptotic cells, preventing secondary necrosis and the release of DAMP factors (79).

Nevertheless, the impact of apoptosis may vary according to cell type and in certain conditions also favor inflammatory responses. Apoptotic endothelial cells externalize phosphatidylserine (80), which binds Factor XII to promote coagulation (81). Apoptotic endothelial cells also interact with other cell types through the release of extracellular vesicles which can in turn promote inflammation. Extracellular vesicles include microvesicles, such as apoptotic bodies, that are produced by cytoplasmic membrane blebbing and shedding, and exosomes, that are smaller and stored in multivesicular bodies or alpha-granules (82). For example, endothelial apoptotic bodies that contain the full-length precursor and processed mature form of IL-1α have pro-inflammatory effects when injected in the peritoneal cavity of mice (83). Both types of vesicles are involved in cellular crosstalk, as will be discussed later.

The relative importance of regulated death pathways in AKI or rejection-induced microvascular injury is only beginning to be unraveled. It is generally accepted that broad caspase inhibition can prevent apoptosis at the expense of increased necroptosis and accentuated renal dysfunction (70, 78), a phenomenon well characterized in renal tubular epithelial cells (84, 85). Cardiac endothelial cells have also been shown to develop RIPK3-dependent cell death after TNF-alpha treatment *in vitro* and following transplantation *in vivo*. RIPK3−/− mice show better preservation of microvascular integrity in a model of cardiac rejection (86). Whether RIPK-dependent death also occurs in the renal microvasculature during AKI and/or rejection remains to be evaluated. However, microvascular apoptosis, evaluated by caspase-3 activation, has been documented in models of renal IRI and rejection (23, 87). Also, inhibition of caspase-3 at the time of renal IRI has generally been associated with improved long-term renal function and reduced extracellular matrix deposition (78, 88, 89). Collectively, these results suggest an important role for caspase-3 in regulating renal vascular cell death whereas the importance of RIPK-dependent death remains to be characterized. While pyroptosis has been observed in renal tubular epithelial cells in a rat model of renal IRI (90), this type of cell death has not been described in endothelial cells.

**Figure 1:**
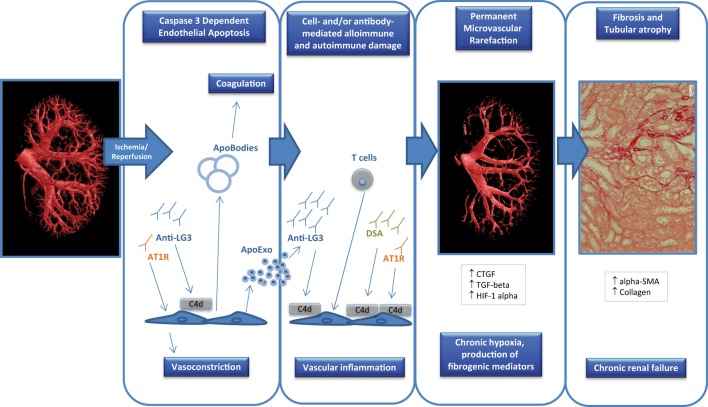
Factors contributing to endothelial dysfunction in transplantation. Ischemia-reperfusion injury that occurs at the time of transplantation can trigger endothelial apoptosis through caspase-3 activation which can in turn release apoptotic bodies with procoagulant activity. In addition, apoptotic endothelial cells also release apoptotic exosome-like vesicles that favor the recruitment of inflammatory cells (e.g., T cells) and the production of autoantibodies such as anti-perlecan/LG3 antibodies (anti-LG3). The synergistic interactions between cellular alloimmunity and autoimmunity, donor-specific antibodies (DSA), and autoantibodies [anti-LG3, anti-angiotensin II type 1 receptors (AT1R)] amplify microvascular damage, through complement-dependent (C4d deposition) or -independent pathways, which leads to permanent peritubular capillary rarefaction. Loss of peritubular capillaries favors chronic hypoxia, leading to overexpression of hypoxia inducible factor 1 α (HIF-1α), favoring transcription of fibrogenic genes such as transforming growth factor β (TGF-β) and connective tissue growth factor (CTGF). It also favors accumulation of collagen, α-smooth muscle actin (α-SMA) positive myofibroblasts and of fibrogenic mediators. These phenomena eventually lead to progressive interstitial fibrosis/tubular atrophy and renal graft dysfunction.

Endothelial caspase-3 activation can promote vascular dysfunction through various and non-mutually exclusive pathways (Figure 1). It favors the release of a number of fibroproliferative mediators, such as CTGF, LG3, and translationally controlled tumor protein, which can in turn favor neointima formation and myointimal thickening (91–94). Endothelial caspase-3 activation also leads to the release of apoptotic bodies or membrane blebs with procoagulant activity (95, 96). Recently, we showed that, in addition to apoptotic bodies, endothelial caspase-3 activation prompts the release of a novel type of extracellular vesicles whose protein content and function are dramatically different from classic apoptotic bodies (97). These apoptotic exosome-like vesicles (ApoExo) are smaller than apoptotic bodies, ranging from 30 to 100 nm and carry active 20S proteasome complexes with pro-inflammatory activity. ApoExo injection in mice favors the recruitment of T and B cells in a model of vascular allograft rejection (97). Endothelial ApoExos also favor the production of autoantibodies such as anti-perlecan/LG3 antibodies, anti-nuclear antibodies, and anti-double-stranded DNA antibodies (97), which in turn further aggravate vascular inflammation. In animal models, renal IRI favors the release of ApoExos within the bloodstream, followed by augmented levels of anti-perlecan/LG3 antibodies (97). Collectively, these results highlight an important role for vascular caspase-3 activation in triggering the release of a number of mediators and extracellular vesicles that will, both at the local and systemic levels, initiate multiple positive feedback mechanisms that favor vascular remodeling, inflammation, and autoimmunity.

## Concluding Remarks

Kidney transplantation is associated with an elevated likelihood of damage to the graft macro- and microvasculature, given the IRI that occurs at the time of transplantation and the physical location of the graft endothelium that makes it a target of choice for cell- or antibody-mediated alloimmune injury. IRI, alloimmune damage, and autoantibodies can activate programmed cell death pathways in the graft endothelium, which can in turn trigger microvascular rarefaction, interstitial fibrosis, and graft dysfunction. These pathways represent potential targets for pharmacological intervention that could be delivered in preservation solutions during the period cold ischemia, with the aim of improving long-term graft outcomes.

## Author Contributions

HC, MD, and M-JH reviewed the literature and drafted the manuscript.

## Conflict of Interest Statement

The authors declare that the research was conducted in the absence of any commercial or financial relationships that could be construed as a potential conflict of interest.

## References

[1] Heitzer T Schlinzig T Krohn K Meinertz T Munzel T. Endothelial dysfunction, oxidative stress, and risk of cardiovascular events in patients with coronary artery disease. Circulation (2001) 104:2673–8. 10.1161/hc4601.099485 11723017

[2] Schachinger V Britten MB Zeiher AM. Prognostic impact of coronary vasodilator dysfunction on adverse long-term outcome of coronary heart disease. Circulation (2000) 101:1899–906. 10.1161/01.CIR.101.16.1899 10779454

[3] Ghiadoni L Cupisti A Huang Y Mattei P Cardinal H Favilla S Endothelial dysfunction and oxidative stress in chronic renal failure. J Nephrol (2004) 17:512–9. 15372412

[4] Cardinal H Raymond MA Hebert MJ Madore F. Uraemic plasma decreases the expression of ABCA1, ABCG1 and cell-cycle genes in human coronary arterial endothelial cells. Nephrol Dial Transplant (2007) 22:409–16. 10.1093/ndt/gfl619 17082211

[5] Amabile N Guerin AP Leroyer A Mallat Z Nguyen C Boddaert J Circulating endothelial microparticles are associated with vascular dysfunction in patients with end-stage renal failure. J Am Soc Nephrol (2005) 16:3381–8. 10.1681/ASN.2005050535 16192427

[6] Faure V Dou L Sabatier F Cerini C Sampol J Berland Y Elevation of circulating endothelial microparticles in patients with chronic renal failure. J Thromb Haemost (2006) 4:566–73. 10.1111/j.1538-7836.2005.01780.x 16405517

[7] Goligorsky MS. Pathogenesis of endothelial cell dysfunction in chronic kidney disease: a retrospective and what the future may hold. Kidney Res Clin Pract (2015) 34:76–82. 10.1016/j.krcp.2015.05.003 26484026 PMC4570605

[8] Laupacis A Keown P Pus N Krueger H Ferguson B Wong C A study of the quality of life and cost-utility of renal transplantation. Kidney Int (1996) 50:235–42. 10.1038/ki.1996.307 8807593

[9] Wolfe RA Ashby VB Milford EL Ojo AO Ettenger RE Agodoa LY Comparison of mortality in all patients on dialysis, patients on dialysis awaiting transplantation, and recipients of a first cadaveric transplant. N Engl J Med (1999) 341:1725–30. 10.1056/NEJM199912023412303 10580071

[10] Kocak H Ceken K Yavuz A Yucel S Gurkan A Erdogan O Effect of renal transplantation on endothelial function in haemodialysis patients. Nephrol Dial Transplant (2006) 21:203–7. 10.1093/ndt/gfi119 16144848

[11] Sharma J Kapoor A Muthu R Prasad N Sinha A Khanna R Assessment of endothelial dysfunction in Asian Indian patients with chronic kidney disease and changes following renal transplantation. Clin Transplant (2014) 28:889–96. 10.1111/ctr.12398 24930933

[12] Olejarz W Bryk D Zapolska-Downar D. Mycophenolate mofetil – a new atheropreventive drug? Acta Pol Pharm (2014) 71:353–61. 25265813

[13] Verhoeven F Prati C Maguin-Gate K Wendling D Demougeot C. Glucocorticoids and endothelial function in inflammatory diseases: focus on rheumatoid arthritis. Arthritis Res Ther (2016) 18:258. 10.1186/s13075-016-1157-0 27814748 PMC5097358

[14] Lamas S Cellular mechanisms of vascular injury mediated by calcineurin inhibitors. Kidney Int (2005) 68:898–907. 10.1111/j.1523-1755.2005.00472.x 16014073

[15] Chapal M Le Borgne F Legendre C Kreis H Mourad G Garrigue V A useful scoring system for the prediction and management of delayed graft function following kidney transplantation from cadaveric donors. Kidney Int (2014) 86:1130–9. 10.1038/ki.2014.188 24897036

[16] Yarlagadda SG Coca SG Garg AX Doshi M Poggio E Marcus RJ Marked variation in the definition and diagnosis of delayed graft function: a systematic review. Nephrol Dial Transplant (2008) 23:2995–3003. 10.1093/ndt/gfn158 18408075 PMC2727302

[17] Coca SG Singanamala S Parikh CR. Chronic kidney disease after acute kidney injury: a systematic review and meta-analysis. Kidney Int (2012) 81:442–8. 10.1038/ki.2011.379 22113526 PMC3788581

[18] Ishani A Xue JL Himmelfarb J Eggers PW Kimmel PL Molitoris BA Acute kidney injury increases risk of ESRD among elderly. J Am Soc Nephrol (2009) 20:223–8. 10.1681/ASN.2007080837 19020007 PMC2615732

[19] Amdur RL Chawla LS Amodeo S Kimmel PL Palant CE. Outcomes following diagnosis of acute renal failure in U.S. veterans: focus on acute tubular necrosis. Kidney Int (2009) 76:1089–97. 10.1038/ki.2009.332 19741590

[20] Lo LJ Go AS Chertow GM McCulloch CE Fan D Ordonez JD Dialysis-requiring acute renal failure increases the risk of progressive chronic kidney disease. Kidney Int (2009) 76:893–9. 10.1038/ki.2009.289 19641480 PMC2771754

[21] Yarlagadda SG Coca SG Formica RN Jr Poggio ED Parikh CR. Association between delayed graft function and allograft and patient survival: a systematic review and meta-analysis. Nephrol Dial Transplant (2009) 24:1039–47. 10.1093/ndt/gfn667 19103734

[22] Basile DP Yoder MC. Renal endothelial dysfunction in acute kidney ischemia reperfusion injury. Cardiovasc Hematol Disord Drug Targets (2014) 14:3–14. 10.2174/1871529X1401140724093505 25088124 PMC4215733

[23] Molitoris BA. Therapeutic translation in acute kidney injury: the epithelial/endothelial axis. J Clin Invest (2014) 124:2355–63. 10.1172/JCI72269 24892710 PMC4089444

[24] Yamamoto T Tada T Brodsky SV Tanaka H Noiri E Kajiya F Intravital videomicroscopy of peritubular capillaries in renal ischemia. Am J Physiol Renal Physiol (2002) 282:F1150–5. 10.1152/ajprenal.00310.2001 11997332

[25] Goligorsky MS Brodsky SV Noiri E. NO bioavailability, endothelial dysfunction, and acute renal failure: new insights into pathophysiology. Semin Nephrol (2004) 24:316–23. 10.1016/j.semnephrol.2004.04.003 15252771

[26] Basile DP Friedrich JL Spahic J Knipe N Mang H Leonard EC Impaired endothelial proliferation and mesenchymal transition contribute to vascular rarefaction following acute kidney injury. Am J Physiol Renal Physiol (2011) 300:F721–33. 10.1152/ajprenal.00546.2010 21123492 PMC3064142

[27] Kwon O Hong SM Sutton TA Temm CJ. Preservation of peritubular capillary endothelial integrity and increasing pericytes may be critical to recovery from postischemic acute kidney injury. Am J Physiol Renal Physiol (2008) 295:F351–9. 10.1152/ajprenal.90276.2008 18562634 PMC2519188

[28] Basile DP Donohoe D Roethe K Osborn JL. Renal ischemic injury results in permanent damage to peritubular capillaries and influences long-term function. Am J Physiol Renal Physiol (2001) 281:F887–99. 10.1152/ajprenal.0050.2001 11592947

[29] Horbelt M Lee SY Mang HE Knipe NL Sado Y Kribben A Acute and chronic microvascular alterations in a mouse model of ischemic acute kidney injury. Am J Physiol Renal Physiol (2007) 293:F688–95. 10.1152/ajprenal.00452.2006 17626153

[30] Basile DP Donohoe DL Roethe K Mattson DL. Chronic renal hypoxia after acute ischemic injury: effects of l-arginine on hypoxia and secondary damage. Am J Physiol Renal Physiol (2003) 284:F338–48. 10.1152/ajprenal.00169.2002 12388385

[31] Basile DP. The endothelial cell in ischemic acute kidney injury: implications for acute and chronic function. Kidney Int (2007) 72:151–6. 10.1038/sj.ki.5002312 17495858

[32] Basile DP. Rarefaction of peritubular capillaries following ischemic acute renal failure: a potential factor predisposing to progressive nephropathy. Curr Opin Nephrol Hypertens (2004) 13:1–7. 10.1097/00041552-200401000-00001 15090853

[33] Steegh FM Gelens MA Nieman FH van Hooff JP Cleutjens JP van Suylen RJ Early loss of peritubular capillaries after kidney transplantation. J Am Soc Nephrol (2011) 22:1024–9. 10.1681/ASN.2010050531 21566051 PMC3374365

[34] Ehling J Babickova J Gremse F Klinkhammer BM Baetke S Knuechel R Quantitative micro-computed tomography imaging of vascular dysfunction in progressive kidney diseases. J Am Soc Nephrol (2016) 27:520–32. 10.1681/ASN.2015020204 26195818 PMC4724942

[35] Babickova J Klinkhammer BM Buhl EM Djudjaj S Hoss M Heymann F Regardless of etiology, progressive renal disease causes ultrastructural and functional alterations of peritubular capillaries. Kidney Int (2017) 91:70–85. 10.1016/j.kint.2016.07.038 27678159

[36] Lim WH Clayton P Wong G Campbell SB Cohney S Russ GR Outcomes of kidney transplantation from older living donors. Transplantation (2013) 95:106–13. 10.1097/TP.0b013e318277b2be 23263504

[37] Slegtenhorst BR Dor FJ Elkhal A Rodriguez H Yang X Edtinger K Mechanisms and consequences of injury and repair in older organ transplants. Transplantation (2014) 97:1091–9. 10.1097/TP.0000000000000072 24646769 PMC4041813

[38] Tasaki M Saito K Nakagawa Y Ikeda M Imai N Narita I Effect of donor-recipient age difference on long-term graft survival in living kidney transplantation. Int Urol Nephrol (2014) 46:1441–6. 10.1007/s11255-014-0655-8 24526331

[39] Oberhuber R Ge X Tullius SG. Donor age-specific injury and immune responses. Am J Transplant (2012) 12:38–42. 10.1111/j.1600-6143.2011.03798.x 22053818

[40] van Willigenburg H de Keizer PLJ de Bruin RWF. Cellular senescence as a therapeutic target to improve renal transplantation outcome. Pharmacol Res (2018) 130:322–30. 10.1016/j.phrs.2018.02.015 29471104

[41] Katsuumi G Shimizu I Yoshida Y Minamino T. Vascular senescence in cardiovascular and metabolic diseases. Front Cardiovasc Med (2018) 5:18. 10.3389/fcvm.2018.00018 29556500 PMC5845435

[42] Clements ME Chaber CJ Ledbetter SR Zuk A. Increased cellular senescence and vascular rarefaction exacerbate the progression of kidney fibrosis in aged mice following transient ischemic injury. PLoS One (2013) 8:e70464. 10.1371/journal.pone.0070464 23940580 PMC3734312

[43] Mueller A Schnuelle P Waldherr R van der Woude FJ. Impact of the Banff ’97 classification for histological diagnosis of rejection on clinical outcome and renal function parameters after kidney transplantation. Transplantation (2000) 69:1123–7. 10.1097/00007890-200003270-00017 10762217

[44] Lefaucheur C Loupy A Vernerey D Duong-Van-Huyen JP Suberbielle C Anglicheau D Antibody-mediated vascular rejection of kidney allografts: a population-based study. Lancet (2013) 381:313–9. 10.1016/S0140-6736(12)61265-3 23182298

[45] Solez K Colvin RB Racusen LC Haas M Sis B Mengel M Banff 07 classification of renal allograft pathology: updates and future directions. Am J Transplant (2008) 8:753–60. 10.1111/j.1600-6143.2008.02159.x 18294345

[46] Nankivell BJ Alexander SI Rejection of the kidney allograft. N Engl J Med (2010) 363:1451–62. 10.1056/NEJMra0902927 20925547

[47] Sis B Einecke G Chang J Hidalgo LG Mengel M Kaplan B Cluster analysis of lesions in nonselected kidney transplant biopsies: microcirculation changes, tubulointerstitial inflammation and scarring. Am J Transplant (2010) 10:421–30. 10.1111/j.1600-6143.2009.02938.x 20055794

[48] Valenzuela NM Mulder A Reed EF HLA class I antibodies trigger increased adherence of monocytes to endothelial cells by eliciting an increase in endothelial P-selectin and, depending on subclass, by engaging FcgammaRs. J Immunol (2013) 190:6635–50. 10.4049/jimmunol.1201434 23690477 PMC3885237

[49] Babu A Andreou A Briggs D Krishnan N Higgins R Mitchell D Clinical relevance of donor-specific IgM antibodies in HLA incompatible renal Transplantation: a retrospective single-center study. Clin Transpl (2016) 32:173–9. 28564535

[50] Everly MJ Rebellato LM Haisch CE Briley KP Bolin P Kendrick WT Impact of IgM and IgG3 anti-HLA alloantibodies in primary renal allograft recipients. Transplantation (2014) 97:494–501. 10.1097/01.TP.0000441362.11232.48 24487396

[51] Herzenberg AM Gill JS Djurdjev O Magil AB. C4d deposition in acute rejection: an independent long-term prognostic factor. J Am Soc Nephrol (2002) 13:234–41. 11752043 10.1681/ASN.V131234

[52] Sis B Jhangri GS Bunnag S Allanach K Kaplan B Halloran PF. Endothelial gene expression in kidney transplants with alloantibody indicates antibody-mediated damage despite lack of C4d staining. Am J Transplant (2009) 9:2312–23. 10.1111/j.1600-6143.2009.02761.x 19681822

[53] Sis B Halloran PF. Endothelial transcripts uncover a previously unknown phenotype: C4d-negative antibody-mediated rejection. Curr Opin Organ Transplant (2010) 15:42–8. 10.1097/MOT.0b013e3283352a50 20009933

[54] Shimizu A Yamada K Sachs DH Colvin RB. Persistent rejection of peritubular capillaries and tubules is associated with progressive interstitial fibrosis. Kidney Int (2002) 61:1867–79. 10.1046/j.1523-1755.2002.00309.x 11967039

[55] Ishii Y Sawada T Kubota K Fuchinoue S Teraoka S Shimizu A. Injury and progressive loss of peritubular capillaries in the development of chronic allograft nephropathy. Kidney Int (2005) 67:321–32. 10.1111/j.1523-1755.2005.00085.x 15610258

[56] Zhang M Alicot EM Carroll MC. Human natural IgM can induce ischemia/reperfusion injury in a murine intestinal model. Mol Immunol (2008) 45:4036–9. 10.1016/j.molimm.2008.06.013 18672288 PMC3230121

[57] Zhang M Alicot EM Chiu I Li J Verna N Vorup-Jensen T Identification of the target self-antigens in reperfusion injury. J Exp Med (2006) 203:141–52. 10.1084/jem.20050390 16390934 PMC2118091

[58] Zhang M Austen WG Jr Chiu I Alicot EM Hung R Ma M Identification of a specific self-reactive IgM antibody that initiates intestinal ischemia/reperfusion injury. Proc Natl Acad Sci U S A (2004) 101:3886–91. 10.1073/pnas.0400347101 14999103 PMC374339

[59] Zhang M Michael LH Grosjean SA Kelly RA Carroll MC Entman ML. The role of natural IgM in myocardial ischemia-reperfusion injury. J Mol Cell Cardiol (2006) 41:62–7. 10.1016/j.yjmcc.2006.02.006 16781728

[60] Yang B Dieude M Hamelin K Henault-Rondeau M Patey N Turgeon J Anti-LG3 antibodies aggravate renal ischemia-reperfusion injury and long-term renal allograft dysfunction. Am J Transplant (2016) 16:3416–29. 10.1111/ajt.13866 27172087

[61] Cardinal H Dieude M Brassard N Qi S Patey N Soulez M Antiperlecan antibodies are novel accelerators of immune-mediated vascular injury. Am J Transplant (2013) 13:861–74. 10.1111/ajt.12168 23432943

[62] Giral M Foucher Y Dufay A Van Huyen JP Renaudin K Moreau A Pretransplant sensitization against angiotensin II type 1 receptor is a risk factor for acute rejection and graft loss. Am J Transplant (2013) 13:2567–76. 10.1111/ajt.12397 23919486

[63] Taniguchi M Rebellato LM Cai J Hopfield J Briley KP Haisch CE Higher risk of kidney graft failure in the presence of anti-angiotensin II type-1 receptor antibodies. Am J Transplant (2013) 13:2577–89. 10.1111/ajt.12395 23941128

[64] Lukitsch I Kehr J Chaykovska L Wallukat G Nieminen-Kelha M Batuman V Renal ischemia and transplantation predispose to vascular constriction mediated by angiotensin II type 1 receptor-activating antibodies. Transplantation (2012) 94:8–13. 10.1097/TP.0b013e3182529bb7 22691955

[65] Nogae S Miyazaki M Kobayashi N Saito T Abe K Saito H Induction of apoptosis in ischemia-reperfusion model of mouse kidney: possible involvement of Fas. J Am Soc Nephrol (1998) 9:620–31. 9555665 10.1681/ASN.V94620

[66] Toronyi E Lord R Bowen ID Perner F Szende B. Renal tubular cell necrosis and apoptosis in transplanted kidneys. Cell Biol Int (2001) 25:267–70. 10.1006/cbir.2000.0620 11352501

[67] Jaffe R Ariel I Beeri R Paltiel O Hiss Y Rosen S Frequent apoptosis in human kidneys after acute renal hypoperfusion. Exp Nephrol (1997) 5:399–403. 9386976

[68] Havasi A Borkan SC. Apoptosis and acute kidney injury. Kidney Int (2011) 80:29–40. 10.1038/ki.2011.120 21562469 PMC4625984

[69] Kishino M Yukawa K Hoshino K Kimura A Shirasawa N Otani H Deletion of the kinase domain in death-associated protein kinase attenuates tubular cell apoptosis in renal ischemia-reperfusion injury. J Am Soc Nephrol (2004) 15:1826–34. 10.1097/01.ASN.0000131527.59781.F2 15213270

[70] Wang S Zhang C Hu L Yang C. Necroptosis in acute kidney injury: a shedding light. Cell Death Dis (2016) 7:e2125. 10.1038/cddis.2016.37 26938298 PMC4823938

[71] Kers J Leemans JC Linkermann A. An overview of pathways of regulated necrosis in acute kidney injury. Semin Nephrol (2016) 36:139–52. 10.1016/j.semnephrol.2016.03.002 27339380

[72] Linkermann A Heller JO Prokai A Weinberg JM De Zen F Himmerkus N The RIP1-kinase inhibitor necrostatin-1 prevents osmotic nephrosis and contrast-induced AKI in mice. J Am Soc Nephrol (2013) 24:1545–57. 10.1681/ASN.2012121169 23833261 PMC3785275

[73] Linkermann A Brasen JH Darding M Jin MK Sanz AB Heller JO Two independent pathways of regulated necrosis mediate ischemia-reperfusion injury. Proc Natl Acad Sci U S A (2013) 110:12024–9. 10.1073/pnas.1305538110 23818611 PMC3718149

[74] Linkermann A. Nonapoptotic cell death in acute kidney injury and transplantation. Kidney Int (2016) 89:46–57. 10.1016/j.kint.2015.10.008 26759047

[75] Linkermann A Chen G Dong G Kunzendorf U Krautwald S Dong Z. Regulated cell death in AKI. J Am Soc Nephrol (2014) 25:2689–701. 10.1681/ASN.2014030262 24925726 PMC4243360

[76] Linkermann A Skouta R Himmerkus N Mulay SR Dewitz C De Zen F Synchronized renal tubular cell death involves ferroptosis. Proc Natl Acad Sci U S A (2014) 111:16836–41. 10.1073/pnas.1415518111 25385600 PMC4250130

[77] Weinlich R Oberst A Beere HM Green DR Necroptosis in development, inflammation and disease. Nat Rev Mol Cell Biol (2017) 18:127–36. 10.1038/nrm.2016.149 27999438

[78] Hébert MJ Jevnikar AM The impact of regulated cell death pathways on alloimmune responses and graft injury. Curr Transplant Rep (2015) 2:242–58. 10.1007/s40472-015-0067-4

[79] Saas P Daguindau E Perruche S. Concise review: apoptotic cell-based therapies-rationale, preclinical results and future clinical developments. Stem Cells (2016) 34:1464–73. 10.1002/stem.2361 27018198

[80] Casciola-Rosen L Rosen A Petri M Schlissel M. Surface blebs on apoptotic cells are sites of enhanced procoagulant activity: implications for coagulation events and antigenic spread in systemic lupus erythematosus. Proc Natl Acad Sci U S A (1996) 93:1624–9. 10.1073/pnas.93.4.1624 8643681 PMC39992

[81] Yang A Chen F He C Zhou J Lu Y Dai J The procoagulant activity of apoptotic cells is mediated by interaction with factor XII. Front Immunol (2017) 8:1188. 10.3389/fimmu.2017.01188 28993777 PMC5622377

[82] Ridger VC Boulanger CM Angelillo-Scherrer A Badimon L Blanc-Brude O Bochaton-Piallat ML Microvesicles in vascular homeostasis and diseases. Position paper of the European Society of Cardiology (ESC) Working Group on atherosclerosis and vascular biology. Thromb Haemost (2017) 117:1296–316. 10.1160/TH16-12-0943 28569921

[83] Berda-Haddad Y Robert S Salers P Zekraoui L Farnarier C Dinarello CA Sterile inflammation of endothelial cell-derived apoptotic bodies is mediated by interleukin-1alpha. Proc Natl Acad Sci U S A (2011) 108:20684–9. 10.1073/pnas.1116848108 22143786 PMC3251090

[84] Lau A Wang S Jiang J Haig A Pavlosky A Linkermann A RIPK3-mediated necroptosis promotes donor kidney inflammatory injury and reduces allograft survival. Am J Transplant (2013) 13:2805–18. 10.1111/ajt.12447 24103001

[85] Linkermann A Hackl MJ Kunzendorf U Walczak H Krautwald S Jevnikar AM. Necroptosis in immunity and ischemia-reperfusion injury. Am J Transplant (2013) 13:2797–804. 10.1111/ajt.12448 24103029

[86] Pavlosky A Lau A Su Y Lian D Huang X Yin Z RIPK3-mediated necroptosis regulates cardiac allograft rejection. Am J Transplant (2014) 14:1778–90. 10.1111/ajt.12779 24984764

[87] Pallet N Dieude M Cailhier J Hebert M. The molecular legacy of apoptosis in transplantation. Am J Transplant (2012) 12:1378–84. 10.1111/j.1600-6143.2012.04015.x 22420581

[88] Zhang X Zheng X Sun H Feng B Chen G Vladau C Prevention of renal ischemic injury by silencing the expression of renal caspase 3 and caspase 8. Transplantation (2006) 82:1728–32. 10.1097/01.tp.0000250764.17636.ba 17198267

[89] Yang C Zhao T Zhao Z Jia Y Li L Zhang Y Serum-stabilized naked caspase-3 siRNA protects autotransplant kidneys in a porcine model. Mol Ther (2014) 22:1817–28. 10.1038/mt.2014.111 24930602 PMC4428396

[90] Yang JR Yao FH Zhang JG Ji ZY Li KL Zhan J Ischemia-reperfusion induces renal tubule pyroptosis via the CHOP-caspase-11 pathway. Am J Physiol Renal Physiol (2014) 306:F75–84. 10.1152/ajprenal.00117.2013 24133119

[91] Sirois I Raymond MA Brassard N Cailhier JF Fedjaev M Hamelin K Caspase-3-dependent export of TCTP: a novel pathway for antiapoptotic intercellular communication. Cell Death Differ (2011) 18:549–62. 10.1038/cdd.2010.126 20966960 PMC3131994

[92] Laplante P Sirois I Raymond MA Kokta V Beliveau A Prat A Caspase-3-mediated secretion of connective tissue growth factor by apoptotic endothelial cells promotes fibrosis. Cell Death Differ (2010) 17:291–303. 10.1038/cdd.2009.124 19730442

[93] Soulez M Pilon EA Dieude M Cardinal H Brassard N Qi S The perlecan fragment LG3 is a novel regulator of obliterative remodeling associated with allograft vascular rejection. Circ Res (2012) 110:94–104. 10.1161/CIRCRESAHA.111.250431 22076637

[94] Pilon EA Dieude M Qi S Hamelin K Pomerleau L Beillevaire D The perlecan fragment LG3 regulates homing of mesenchymal stem cells and neointima formation during vascular rejection. Am J Transplant (2015) 15:1205–18. 10.1111/ajt.13119 25808553

[95] Gao C Xie R Yu C Ma R Dong W Meng H Thrombotic role of blood and endothelial cells in uremia through phosphatidylserine exposure and microparticle release. PLoS One (2015) 10:e0142835. 10.1371/journal.pone.0142835 26580207 PMC4646287

[96] Dignat-George F Boulanger CM. The many faces of endothelial microparticles. Arterioscler Thromb Vasc Biol (2011) 31:27–33. 10.1161/ATVBAHA.110.218123 21160065

[97] Dieude M Bell C Turgeon J Beillevaire D Pomerleau L Yang B The 20S proteasome core, active within apoptotic exosome-like vesicles, induces autoantibody production and accelerates rejection. Sci Transl Med (2015) 7:318ra200 10.1126/scitranslmed.aac9816 26676607

